# Supplementary material for: Dietary Habits Are Related to Phase Angle in Male Patients with Non-Small-Cell Lung Cancer
Source: Curr Oncol. 2022 Oct 26;29(11):8074–83. doi: 10.3390/curroncol29110637 (PMC9689548; doi:10.3390/curroncol29110637)
Supplement: Supplementary file 1 [file curroncol-29-00637-s001.zip › curroncol-1926467-supplementary.pdf]

Supplementary Table S1. Component loadings derived using Principal Component Analysis for the identification of dietary patterns.

	1	2	3	4
	**Whole grains, fruits, vegetables**	**Potatoes, meat, poultry**	**High olive oil, low alcohol**	**Legumes, fish**
**Whole wheat grains (portions/ week)**	**0.559**	0.352	0.016	0.005
**Potatoes (portions/week)**	0.018	**0.609**	0.220	0.265
**Fruit (portions/week)**	**0.703**	-0.149	0.247	-0.018
**Vegetables (portions/week)**	**0.832**	0.048	-0.184	0.062
**Legumes (portions/week)**	-0.027	0.098	-0.167	**0.729**
**Fish (portions/week)**	0.083	-0.050	0.131	**0.720**
**Meat (portions/week)**	-0.083	**0.607**	-0.059	-0.395
**Poultry (portions/week)**	0.075	**0.729**	-0.017	0.017
**Dairy (portions/week)**	0.384	-0.248	0.440	0.166
**Olive oil (portions/week)**	0.093	0.101	**0.696**	-0.280
**Alcohol (portions/week)**	0.085	-0.083	**-0.741**	-0.136
**% Cumulative variance explained**	16.8	31.0	43.2	54.7

Higher absolute values of the loadings indicate that the food is correlated with the respective component. Numbers in bold indicate absolute loadings greater than 0.5, which correspond to a specific component.
